# Successful Oral Immunotherapy (OIT) Due to Anti-IgE Protection

**DOI:** 10.3390/jcm14186612

**Published:** 2025-09-19

**Authors:** Mehrak Yoosefi Moridani, Susanne Lau, Kirsten Beyer

**Affiliations:** 1 Praxis für Kinderpneumologie/Allergologie, 10625 Berlin, Germany; mehrak.yoosefi@gmx.de; 2 Department of Pediatric Respiratory Medicine, Immunology and Critical Care Medicine, Charité Universitätsmedizin Berlin, 13353 Berlin, Germany; susanne.lau@charite.de; 3 German Center for Child and Adolescent Health (DZKJ), Partner Site Berlin, 13353 Berlin, Germany

**Keywords:** peanut allergy, oral immunotherapy, anti-IgE, omalizumab

## Abstract

**Background/Objectives**: Oral immunotherapy with peanut protein powder is difficult to initiate in patients with a very low reactivity threshold to peanuts. For this specific group, an add-on treatment with omalizumab (anti-IgE monoclonal antibody) is helpful to tolerate the initial steps. **Methods**: After failed initiation in three children, an off-label approval from the individuals’ insurance was necessary for the premedication with anti-IgE ahead of a second approach at our center. Currently, the European countries have no approval from the European Medicines Agency (EMA) for the treatment of food allergies with omalizumab. **Results**: Under anti-IgE protection, our patients have restarted the oral immunotherapy without anaphylactic reactions and have reached the maintenance dose within the treatment protocol. **Conclusions**: For a few patients with initial anaphylactic reactions due to doses below the first treatment step (3 mg peanut protein powder), a premedication with omalizumab is not only safe but also reduces side effects, in particular anaphylactic reactions, and the time to reach the hundredfold higher maintenance dose (300 mg peanut protein powder).

## 1. Introduction

Peanut allergy treatment historically consisted of allergen avoidance until the approval of an oral immunotherapy (OIT) for children aged 4–17 years by the U.S. Food and Drug Administration (FDA) and the European Medicines Agency (EMA) in 2020 [1,2,3,4,5,6]. The aim of an oral immunotherapy is induction of desensitization, increase in reaction threshold and protection in case of accidental ingestion, hence a modification of the natural history of the culprit food [1,4,7,8,9]. However, peanut oral immunotherapy has limitations due to adverse reactions, which may lead to non-compliance and discontinuation [1,8]. Therefore, the FDA approval of omalizumab (anti-IgE monoclonal antibody) for the reduction in allergic reactions that may occur with an accidental exposure to one or more foods in adults and children aged 1 year and older with IgE-mediated food allergy in February 2024 marks a milestone in the treatment of food allergies [10,11]. Despite the data of the phase 3 clinical trial, Omalizumab as Therapy in Children and Adults (OUtMATCH), and the US approval, there is currently no indication for the treatment of IgE-mediated food allergies in Europe [10,12,13].

Food-induced fatal anaphylactic reactions are very rare in children, despite their highest rate of anaphylaxis compared with other age groups [11,14]. Most of the anaphylactic reactions occur outside the home [14]; therefore, patients and caregivers should be instructed to recognize and treat those reactions [15,16]. In European countries including Germany, the only available in-label treatment that could modify the anaphylactic risk of patients with a food allergy to peanut is oral immunotherapy [14]. The identification of patients at greatest risk of more severe allergic reactions is not yet reliably possible [11]. While on oral immunotherapy, the highest rate of allergic reactions occurs during the initial build up phase; mainly mild to moderate symptoms are recorded [17]. Providers of an OIT should therefore offer appropriate equipment and infrastructure to accompany the treatment, in areas with limited access under close supervision by a qualified allergist [16]. For a secured intake, “safe-dosing” rules should be shared with patients and their caregivers, including an individualized dosing in case of augmentations factors such as physical activity, hot showers, infections, intake of nonsteroidal anti-inflammatory medication or sleep deprivation [15,18].

In the food allergy management, omalizumab offers several beneficial options by reducing free IgE in the blood, especially a decreased risk of anaphylaxis when exposed to the allergen [19]. Used as a monotherapy, this anti-IgE biologic effects the threshold of the reactivity dose to the culprit food, whereas an add-on treatment to an oral immunotherapy shows a positive effect for achieving rapid desensitization in a safe and effective manner [15,19,20]. The possibilities for food allergy management (avoidance, oral immunotherapy, omalizumab) should always be discussed with patients and their caregivers in a shared decision-making process to plan an individualized strategy [17].

## 2. Materials and Methods

Oral immunotherapy (OIT) with peanut protein powder in children was first started in summer 2022 on the pediatric ward of Charité Universitätsmedizin Berlin (Campus Virchow Klinikum), due to the pandemic situation and shortness of required capacity.

An inpatient admission was necessary for the initiation of the treatment. According to the medication guide of Palforzia^TM^ (Peanut (Arachis hypogaea) Allergen Powder-dnfp, Aimmune Therapeutics^TM^, Inc., Brisbane, CA, USA) the initial five escalation doses (0.5 mg to 6 mg) were planned to be administered on a single day under allergist supervision. In our center, a complete physical check-up was administered before the start of the treatment, an individualized emergency plan was set up and parental written consent was obtained. After tolerating the first steps, the up-dosing phase—starting with 3 mg peanut protein powder—could continue on the following day.

Two of the children from our center and one external patient had an anaphylactic reaction during the initial build up phase and consequently OIT was discontinued. Because the reactions occurred to doses lower or equal to the first step (3 mg peanut protein powder), parental consent was mandatory for further action. A multidisciplinary team including allergists and specialized dietitians discussed the three cases separately, at the given time. The caregivers were informed about the possibility of an add-on treatment to oral immunotherapy as a second chance to induce a stable reactivity threshold to a significantly larger amount of peanut protein.

A PubMed-based (PubMed, MEDLINE; pubmed.ncbi.nlm.nih.gov) research was performed between 2 November 2023 and 8 November 2023 using the keywords “peanut”, “oral immunotherapy”, “omalizumab”, “food allergy”, “anaphylaxis” and “children”. The available studies were searched for relevance; a total of 15 articles in English language were considered as appropriate to the topic. A second literature search was accomplished in June 2024, half a year after the first attempt, and another nine relevant studies were detected.

Next, an individual off-label approval from the patients’ insurance was obtained and after agreement a premedication with omalizumab (Xolair^®^, Novartis Pharma, Nürnberg, Germany) ahead of a second initiation of OIT was started.

Laboratory examinations were performed at least prior to the first treatment approach in our center; the reference values of the local laboratory were used. Serum total immunoglobulin (IgE) and specific IgE were tested with an immunoassay (Phadia, Thermo Fisher Scientific, Uppsala, Sweden).

This retrospective study was approved by the Ethik-Kommission Campus Virchow of Charité Universitätsmedizin, Berlin.

## 3. Results

### 3.1. Patients

The treatment with peanut protein powder (Arachis hypogaea Allergen Powder-dnfp; PTAH; AR 101; Palforzia^TM^, Aimmune Therapeutics, Inc., Brisbane, CA, USA) was started in summer 2022 in our pediatric department in Charité Universitätsmedizin, Berlin. The eligible patients were either from our own center or sent from an external pediatrician, from Berlin or the surrounding areas. External patients were initially seen in our outpatient pediatric allergy department, and the indication for the treatment was confirmed by a specialized allergist. Within the first two years, we started OIT in nearly fifty patients of which only two could not successfully be initiated (Table 1: patients no. 1 and 3).

Our first patient is very well known in our pediatric allergy department. Due to an early manifestation of atopic dermatitis, he was regularly seen in our outpatient clinic. Because of several sensitizations to food allergens, we performed oral food challenges in the past, starting at ten months of age with cow’s milk, soy and wheat. Altogether, five planned inpatient admissions for food challenge were performed in five years, with a total of nine allergens including repeated provocations. He built up natural tolerances against hen’s egg, cow’s milk, soy and wheat within these years and showed sensitizations without clinical relevance against hazelnut and cashew and pistachio, respectively. At the age of five years, only two positive provocations were remaining: against walnut and pumpkin seed. The first and only recorded reaction to peanuts was at the age of 3 years. He developed hives all over his body. Further laboratory tests were performed to examine related legumes; he was sensitized to pea, chickpea and lentil, but not to white beans.

The second patient was sent to our outpatient clinic by his pediatrician from outside of Berlin (distance to our clinic: 262 km). He had repeated accidental ingestions of products containing peanut; first at the age of 4 years, then three times in the following years. His last known reaction was at the age of eight years with immediate oral itchiness and tightness in his throat. He has no other food allergies and regularly consumes legumes and tree nuts.

The parents of the third patient have made an appointment in our outpatient clinic to discuss the treatment options for their daughter. She had first reacted at the age of two years; two further reactions occurred in the following six months. Every reaction followed the same pattern: conjunctival reaction, peroral itchiness, swelling of the tongue as well as hoarseness and coughing. She is mono-sensitized to peanuts and has atopic dermatitis. During the winter months her skin is more affected with dryness and itchiness.

None of our three patients has asthma as an atopic comorbidity.

### 3.2. First Oral Immunotherapie (OIT) Patients

After written consent from the parents, OIT was scheduled. Generally, all patients under 10 years of age were planned for inpatient admission. Therefore, our patients were scheduled by our dietician and attended our ward with at least one parental accompaniment. The initiation of OIT took part on two days; the up-dosing is according to the protocol of the used peanut protein powder (Palforzia^TM^) and under pediatrician supervision. Ahead of the start, our patients were checked for infections, and for safety reasons an intravenous peripheral cannula was inserted. In this context, we tested total IgE and specific IgE for peanuts and Ara h 2 prior to treatment (Table 2: patients 1 and 3).

Compared with the first ever tested concentrations of total Immunoglobulin E (IgE) and specific IgE for peanut, the levels were significantly higher (Table 2: patient 1, 12-fold higher total IgE and patient 3, 6.7-fold, respectively). At admission, the serum concentration of the major peanut component Ara h 2 was above the cut-off of 42.2 kU/L, a predictor of clinically relevant peanut allergy, as previously shown [21].

We successfully initiated oral immunotherapy in 28 patients before patient no. 1 reacted in November 2023 (Table 3). His reaction occurred after the fourth dose (3 mg peanut protein powder) of Palforzia^TM^; he complained about itchiness of the skin and developed urticaria, vomiting, coughing and bronchospasm. He received epinephrine (adrenaline autoinjector 150 µg) intramuscularly as well as antihistamine (clemastine), prednisolone and a balanced electrolyte solution intravenously. Within a few minutes the reaction was stopped and the symptoms resolved.

In another German clinic, patient no. 2 reacted after 1.5 mg peanut protein powder (3rd dose of Palforzia^TM^) in October 2023 with initial oral allergy symptoms and subsequently bronchospasm. He was treated with epinephrine inhalation besides prednisolone intravenously and antihistamine (dimetindene) orally. After this treatment, his symptoms were quickly resolved. In the clinic, the family was informed about further options such as combination therapy with a biologic agent. Together with their outpatient pediatrician, they decided to contact our allergy department for further support.

Our youngest patient so far started oral immunotherapy a few weeks after reaching the age of four years. Her reaction happened at the dosage of 3 mg peanut protein powder (4th dose of Palforzia^TM^) in August 2024, with anaphylactic symptoms including itchiness, coughing and bronchospasm. Her treatment consisted of epinephrine intramuscularly, antihistamine (cetirizine) orally and prednisolone intravenously. After the reaction, she fell asleep; therefore, further therapy options were discussed.

All three patients were treated with epinephrine, antihistamine and prednisolone, additionally patient no. 1 received the volume intravenously due to circulation problems. All OIT treatments were consequently discontinued after failed initiation. The parents of all patients were willing to undergo a second attempt of oral immunotherapy with the same peanut protein powder under comedication with anti-IgE.

### 3.3. Pretreatment with Omalizuab

Within our allergy department, we discussed the treatment options for these vulnerable children who needed extra protection regarding their very low reactivity threshold to peanuts. Without European approval by EMA for omalizumab (anti-IgE monoclonal antibody) for this indication, we could not offer an on-label add-on treatment. After parental consent and the literature research, we, therefore, prepared individual off-label requests to the responsible patients’ insurance, containing the most recent study results and promising articles as well as the available U.S. approval for this biologic agent for the indication of food allergy. Only for the first off-label request from our center, the insurance company demanded additional information on an extra internal company paper, including expected duration and costs of the treatment. We received approvals after approximately 8 weeks for all three patients.

The insurance of our own patients authorized the medication with omalizumab (Xolair^®^) for 6 months, whereas our external patient was initially granted 12 months. The following requests for continuation of the treatment with information about the then succeeded steps were without any further problems or delays.

According to the then published studies, we started premedication with omalizumab subcutaneously, independent from the patients’ total IgE and body weight (see Table 4).

For each patient, appointments were planned in our pediatric allergy outpatient clinic as soon as the off-label approval arrived for the fastest possible start. After written parental consent, the injections of omalizumab (two prefilled syringes à 150 µg, Xolair^®^) were administered, one in each upper arm subcutaneously. The injections were given by a pediatric allergist, familiar with allergen immunotherapy administration subcutaneously and anaphylactic reactions. The batch numbers of the syringes were recorded in the patients’ outpatient file, in case of adverse events. The second dose (2 × 150 µg s.c.) was planned exactly four weeks after the first injections. Our first patient decided to use plasters with local anesthetic cream (lidocaine and prilocaine, EMLA^®^, Aspen Pharma Trading Limited, Dublin, Ireland) to reduce the injection pain. They were placed in the right position, either by a doctor or later on by his mother.

The duration of the premedication (8 weeks) was determined at the start, according to the then available papers about omalizumab and oral immunotherapy.

### 3.4. Second Oral Immunotherapy

Synchronized with the third injection of omalizumab, after 8 weeks of the start of the biologic treatment (week 0 + week 4), the second attempt of OIT was initialized at our pediatric ward. After a physical check-up, an emergency medication plan was set up and an intravenous cannula was inserted. With the latter, another blood test was performed to monitor the process. As expected, the total IgE levels were further elevated due to the anti-IgE medication (Table 5). Because of the previous experiences with the failed initiation, an additional pediatric allergist was present during this start. With the help of one specialized dietician, the first steps were administered in the regular pattern. After tolerating the first dose, no extra time was needed for security reasons. Every thirty minutes, the next portion was consumed. All three patients tolerated the initial up-dosing (five doses of Palforzia^TM^) without any side effects. Therefore, the further build-up phase could be planned as outpatient admissions under supervision.

The time until the maintenance dose was within the normal variations in the treatment (patients no. 1 and no. 3) in our center. Only the second patient continued the further up-dosing externally and local effects of the injection (pain, swelling, redness of the skin) as well as abdominal pain and nausea after the intake of the peanut protein powder have been reported. This patient’s parents have chosen to complete the build-up treatment close to their home, we therefore guaranteed 24/7 availability per mail and telephone for both the parents and the supervising pediatrician.

### 3.5. Omalizumab and OIT

**Figure 1:**
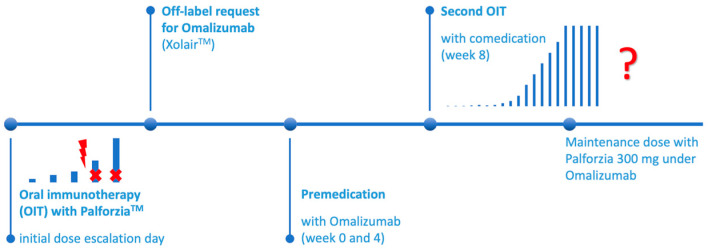
Timeline. The question mark relates to the still unkown length of treatment with omalizumab after the build-up phase of OIT.

Every second week, the up-dosing of the peanut protein powder was planned in our outpatient pediatric allergy center, every fourth week at the same time with the omalizumab injections. The approval for the anti-IgE treatment lasted for only 6 and 12 months, respectively; hence, at least another off-label permission was necessary to guarantee a safe management of OIT until the maintenance dose (Figure 1). Due to a lack of studies regarding this topic, we started to reduce the dosage of omalizumab in patient no. 1 after three months on the maintenance dose, from 300 mg Xolair^®^ subcutaneously to 150 mg s.c. while keeping the dosage of Palforzia^TM^ on the same level. In the future, a termination of omalizumab is planned after several months during the maintenance dose, presuming that the patients have built up enough protecting IgG_4_ against peanut to safely tolerate the amount of 300 mg peanut protein powder and one peanut kernel, respectively.

## 4. Discussion

A very small percentage of children eligible for oral immunotherapy with peanut protein powder has anaphylactic reaction to minimal dosages during initiation, in our center less than 5% of the patients treated with Palforzia^TM^. These patients are at incredible high risk for accidental ingestion and severe anaphylactic reactions in real life due to contamination and potential accidental exposure to products containing peanuts. In our small cohort, none of the three patients suffered from asthma; therefore, one major risk factor for anaphylactic reactions was absent.

Recently the age limit for the initiation of peanut OIT was lowered to one year of age; this will also modify the disease’s history [22]. Starting a daily treatment at an early age might not only lower the risk of adverse reactions and improve the quality of life, it also has a potential for long-term remission, behavioral adaptability and is beneficial due to an enhanced immune system plasticity [23]. We expect a higher acceptance of the peanut protein powder in this age group, less psychological problems with the intake but an extended up-dosing phase due to febrile infections, hence a more individualized build-up phase.

In order to reduce unforced adverse events, every patient in our department is examined prior to OIT to exclude infections as one of the augmentation factors. Ahead of treatment, the caregivers and medical staff need to be informed about further trigger factors and need to be trained in emergency treatment in case of an anaphylactic reaction to minimize the patient’s risk during the treatment.

In our center, only a few patients discontinued their treatment due to adverse reactions (in total less than 20%) during the build-up phase; mainly, abdominal problems were named as reasons for stopping the treatment. These patient groups, in particular teenagers, need to be studied in future for the combination treatment of omalizumab and OIT. Our three treated patients were younger than teens; nonetheless, the oldest one did suffer from side effects despite the anti-IgE comedication. Teenagers in particular have an increased risk of accidental reactions due to a lack of parental supervision and their possibly risky behavior outside home in peer groups. These children, especially, require safe options to build up a tolerance to at least one peanut and the maintenance dose of OIT, respectively, to be protected in case of accidental ingestion.

The aim of the temporary comedication of omalizumab during the oral immunotherapy is to build up enough protecting IgG4 antibodies to build up a stable higher reactivity threshold or even a tolerance.

In Germany, an add-on treatment with omalizumab (anti-IgE) is not (yet) approved but with the FDA approval and data from the OUtMATCH study [10,13,24], an individual off-label treatment was granted by the insurance after written requests. The three different insurance companies each approved not only the first request but also every following to continue the biologic comedication. Hence, no delays or restrictions occurred during the treatment period. Our campus pharmacy provided us with the necessary medication, rapidly after each order. In our outpatient clinic, there was enough capacity to store the packages according to the medications’ requirements.

In our group of children, only one of the patients suffers from additional food allergies. The impact of omalizumab on those allergens was not studied during his treatment. Further on, more studies are necessary for multi-OIT under anti-IgE protection to maximize the success of OIT treatments.

At the start of the first treatment with omalizumab the available papers recommended using a standard dosage of 300 mg subcutaneously every four weeks, comparable to the treatment of chronic spontaneous urticaria. Recently, a consensus paper published a dosing nomogram for IgE-mediated food allergies regarding the omalizumab dose, dependent on pretreatment Serum IgE and body weight [25]. With knowledge of this data, our first patient received the right dosage but not every two weeks, whereas the second patient received the right dosage in the correct interval. Due to the lack of pretreatment laboratory results, no clear statement is possible for our youngest patient. Using her older total IgE values, her correct dose might have been lower (225 mg instead of 300 mg) but presuming an increase in her total IgE level over time, possibly the used amount was also correct. In the future, an even more personalized treatment with this biologic agent will be possible.

Missing data regarding the end of the treatment with omalizumab in food allergic children led to our approach of treatment tapering, mainly because of security aspects. The duration of the full dose of omalizumab was according to the OUtMATCH study. The tapering strategy was discussed for chronic spontaneous urticaria and omalizumab at that time [26]. Limitations of our paper are the very small number of patients reported and the retrospective collection of data. At the start of our treatment, the final results from the OUtMATCH study were not yet published and the duration of the combination therapy was not determined. Generally, the anti-IgE treatment is planned for a long-term duration because the effects fade off with discontinuation. The FDA approval for the indication food allergy states a conjunction with allergen avoidance [17].

Currently, studies are testing the effects of other biologic agents, such as dupilumab in a phase II, multicenter, open-label study [5,27]. Dupilumab is currently approved for the treatment of severe atopic dermatitis in infants from 6 months of age in Germany. This treatment option may possibly alter the development of atopic multi-morbidities in these children and also modify their possible food allergy history due to an improvement in the skin barrier disorder as an otherwise possible entrance for sensitizations towards foods. The treatment with dupilumab for infants with severe atopic dermatitis might be beneficial for only those patients developing IgE-mediated food allergies through epicutaneous sensitization [28] due to barrier dysfunction of the skin. However, the dual-allergen hypothesis states other sensitization paths like through the airway or in utero; these patients might still develop food allergies despite the biologic treatment [28]. Furthermore, it is unknown how long a treatment with a biologic like dupilumab has to be performed in order to avoid further atopic manifestations. Although IgE to food allergens decreases during treatment with dupilumab, the clinical reaction to foods in challenge tests is not necessarily blocked [29].

In the future, the correct pre-selection of children suffering from food allergies will be of crucial importance. Prescribing omalizumab in those children being at high risk due to low reaction thresholds and anticipating a high-risk situation causing anxiety in affected patients or their caregivers, respectively, might serve as a potential bridge to OIT [30]. Also, the presence of comorbidities like allergic asthma, chronic rhinosinusitis with nasal polyps and chronic spontaneous urticaria should be considered, as these patients with multiple allergic disorders would profit from a single medication [17]. As a preferred option, omalizumab should be offered to patients with the desire of a non-daily or non-oral treatment, to those failing previous immunotherapy and to those with multiple food allergies [17].

Further studies should evaluate outcome parameters like sustained unresponsiveness, remission, and the ratio between costs and benefit, respectively. Every patient should be offered an individualized treatment plan; shared decision-making should always be part of the process. Further studies and real-life data with higher numbers of patients are essential to optimize a treatment scheme with omalizumab add-on to the food OIT including a reduction scheme once the maintenance dose is reached, without putting those patients in danger for the remaining time of the treatment. The specialized knowledge of the university departments should be accessible for outpatient facilities, so that more affected children can benefit from existing therapeutic options.

## Figures and Tables

**Table 1:** Epidemiological data.

	No. 1	No. 2	No. 3
Sex	male	male	female
Age at first OIT *	6 years	9 years	4 years
Weight at first OIT *	21 kg	30 kg	16 kg
First known reaction to peanut (age)	3 years	4 years	2 years
Sensitization to other foods	yes	no	no
Other atopic comorbidities	yes (atopic dermatitis)	yes (sensitization to inhalative allergens)	yes (atopic dermatitis)
Family history of atopic diseases	positive	negative	positive

* OIT = oral immunotherapy.

**Table 2:** Laboratory testing.

	No. 1	No. 2	No. 3
First testing at the age of - Total IgE - sIgE peanut (CAP class) - sIgE Ara h 2	1 year 128 kU/L 13.4 kU/L (3) NA	6 years 531 IU/mL * > 100 kU/L (6) * > 100 kU/L (6) *	2 years 110 IU/mL * 15.7 kU/L (3) * NA
Testing prior first OIT - Total IgE - sIgE peanut - sIgE Ara h 2	1535 kU/L >100 kU/L (6) >100 kU/L (6)	NA NA NA	737 kU/L 58.7 kU/L (5) 33.6 kU/L (4)

* external laboratory testing; sIgE = specific Immunoglobulin E; CAP = carrier-polymere-system; Ara h 2 = Arachis hypogaea 2; NA = not available (not measured).

**Table 3:** First oral immunotherapy attempt.

	No. 1	No. 2	No. 3
Reaction to dose (peanut protein powder) Severity grade of reaction (Ring & Messner)	3 mg III	1.5 mg III	3 mg III
Treatment - epinephrine - antihistamine - prednisolone - infusion	i.m. i.v. i.v. yes	inh. p.o. i.v. no	i.m. p.o. i.v. no

i.m. = intramuscular, inh. = inhalative, i.v. = intravenous, p.o. = per os.

**Table 4:** Treatment with omalizumab.

	No. 1	No. 2	No. 3
Dosage per injection Interval of injections Pretreatment prior to 2nd OIT	300 mg s.c. every 4 weeks eight weeks	300 mg s.c. every 4 weeks eight weeks	300 mg s.c. every 4 weeks eight weeks
Laboratory results before treatment - Total IgE - sIgE peanut - sIgE Ara h 2	1787 kU/L >100 kU/L (6) >100 kU/L (6)	316 kU/L >100 kU/L (6) >100 kU/L (6)	NA NA NA

s.c. = subcutaneous; NA = not available (not measured).

**Table 5:** Second OIT.

	No. 1	No. 2	No. 3
initiation time until maintenance dose adverse events during build-up local injection reactions	successful 28 weeks none no	successful >22 weeks * mild yes	successful 22 weeks none no
Testing prior to second OIT - Total IgE - sIgE peanut - sIgE Ara h 2	4624 kU/L >100 kU/L (6) >100 kU/L (6)	1951 kU/L >100 kU/L (6) >100 kU/L (6)	NA NA NA

* external continuation; NA = not available (not measured).

## Data Availability

The original contributions presented in this paper are included in the article. Further inquiries can be directed at the corresponding author.

## Abbreviations

The following abbreviations are used in this manuscript:

OIT	Oral immunotherapy
IgE	Immunoglobulin E
EMA	European Medicines Agency
FDA	Food and Drug Administration
s.c.	Subcutaneous
IgG_4_	Immunoglobulin G_4_
